# Strontium Salicylate

**Published:** 1916-03

**Authors:** 


**Strontium Salicylate** is declared, editorially, Jour. A. M. A., Jan. 29, not to be superior to sodium-salicylate and, indeed, to furnish only 4-5 of the salicylic radicle available from the latter. This is in support of an original article by M. A. Blankenhorn of Chicago in the same issue. A table is presented, mainly of rheumatic and acute endocarditic cases. (Note: While a clinical study of this nature is of great value, it does not settle the question. Strontium salicylate has, in several cases in our experience, been followed with a marked and prolonged diminution of sugar, even on a fair allowance of carbohydrates, and it has seemed that strontium bromid has had a more lasting sedative action than sodium bromid. Clinical tests are quite apt to be misleading on either side of a question. Our impression as to the value of strontium, in and of itself, may be mistaken, but it is premature to dismiss this drug without further investigation, especially as the authority for its use is considerable. As to the class of cases with which Blankenhorn deals, we are quite ready to admit that the essential therapeutic radicle in rheumatic affections is the salicylic and that if any advantage rests with any particular metallic base, it is with those that are markedly alkaline, although superacidity in the direct sense is not so marked and so general in what we term rheumatism, as is often omagined. —Ed.).

